# DHODH tangoing with GPX4 on the ferroptotic stage

**DOI:** 10.1038/s41392-021-00656-7

**Published:** 2021-06-18

**Authors:** Fudi Wang, Junxia Min

**Affiliations:** 1 The First Affiliated Hospital, The Second Affiliated Hospital, Hengyang Medical School, University of South China, Hengyang, China; 2 The First Affiliated Hospital, Institute of Translational Medicine, Cancer Center, School of Public Health, Zhejiang University School of Medicine, Hangzhou, China

**Keywords:** Cell biology, Molecular biology

Ferroptosis, an iron-dependent form of regulated cell death, is prevented by activity of the glutathione-dependent phospholipid hydroperoxidase GPX4 (Glutathione peroxidase 4) in the cytosol and mitochondria, and by the glutathione-independent CoQ10 oxidoreductase FSP1 at the plasma membrane. In their recent paper published in *Nature*, Mao et al. report that DHODH (Dihydroorotate dehydrogenase) coordinates with GPX4 to block ferroptosis in the mitochondrial inner membrane by reducing ubiquinone to form ubiquinol in cancer cells, thus providing a novel targeted strategy for treating cancer.^1^

**Fig. 1:**
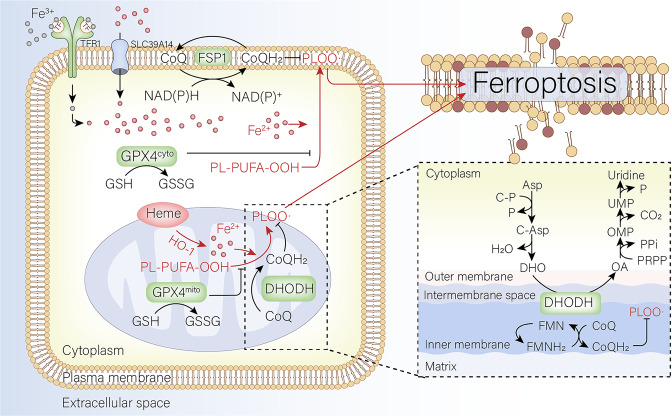
Schematic model depicting the various pathways in the plasma membrane, cytoplasm, and mitochondria that lead to ferroptosis. Ferroptosis is characterized by iron-dependent cell death with accumulated PLOO. Iron uptake is controlled by TFR1-mediated diferric (Fe3+) transferrin-bound iron and SLC39A14-mediated non-transferrin-bound iron (Fe2+). Whereas PLOO-induced ferroptosis is regulated by either GSH-dependent GPX4 in the cytosol and mitochondria or GSH-independent FSP1 at the plasma membrane. Mao et al. identified DHODH coordinates with GPX4 to inhibit ferroptosis in the mitochondrial inner membrane. PLOO phospholipid hydroperoxyl radical, TFR1 transferrin receptor 1, GSH glutathione, GPX4 glutathione peroxidase 4, FSP1 ferroptosis suppressor protein 1, DHODH dihydroorotate dehydrogenase, CoA coenzyme A, PL-PUFA-OOH phospholipid-polyunsaturated fatty acid-hydroperoxide, GSSG oxidized glutathione, NAD(P)H reduced nicotinamide adenine dinucleotide (phosphate), NAD(P)+ oxidized nicotinamide adenine dinucleotide (phosphate)

Ferroptosis is an iron-dependent, non-apoptotic form of regulated cell death involving lipid peroxidation.^2^ Although the physiological role of ferroptosis has yet to be identified, its pathological functions have been documented in diseases affecting a variety of organs, including the heart, liver, kidney, brain, etc.^2,3^ Notably, compelling evidence supports the notion that targeting genes and pathways that inhibit ferroptosis may provide new opportunities for treating a variety of cancers.^2^ In the past decade, two ferroptosis-inhibiting mechanisms have been identified, involving a wide range of genes and signaling pathways, including the GPX4-driven glutathione-dependent and FSP1-driven CoQ10-dependent metabolic pathways. Importantly, Mao et al. have now linked the mitochondrial enzyme DHODH (dihydroorotate dehydrogenase) to ferroptosis by performing metabolomics analyses in cancer cells treated with the GPX4 inhibitors RSL3 and ML162 to induce ferroptosis (see Fig. 1).^1^

As an iron-containing flavin-dependent enzyme, DHODH plays an essential role in the *de novo* synthesis of pyrimidines. DHODH catalyzes the conversion of dihydroorotate to orotate via a redox reaction; orotate is then converted to uridine monophosphate, the RNA nucleotide involved in ribosome biogenesis. Based on its essential roles in pyrimidine synthesis and mitochondrial function, DHODH inhibitors were originally used in the treatment of rheumatoid arthritis and multiple sclerosis. Over half a century ago, the expression and enzymatic activity of DHODH were first associated with cancer progression, yet emerged only recently as a potential therapeutic target for cancer. Since then, considerable effort has been invested in developing DHODH inhibitors and determining their efficacy as well as their anti-cancer and anti-viral potential. However, the mechanisms underlying this enzyme’s role in various malignancies and other diseases are poorly understood. To this end, Mao et al. show that ferroptosis is the key mechanism by which inhibiting DHODH exerts its anti-cancer activity. Their findings suggest that DHODH and mitochondrial GPX4 are two key metabolic enzymes that function to detoxify lipid peroxides that accumulate in the mitochondria.^1^ Thus, inhibiting both DHODH and GPX4 promotes ferroptosis by increasing lipid peroxidation in the mitochondria, providing a promising strategy for targeting mitochondrial DHODH and GPX4 in cancer cells. In addition, Mao et al.’s data provide compelling evidence that DHODH inhibitors may be used to treat GPX4^low^ cancers; moreover, DHODH inhibitors may be combined with sulfasalazine (an inducer of ferroptosis that inhibits the cystine/glutamate antiporter xCT (SLC7A11/SLC3A2 complex), a component of system x_c_^-^) in order to treat GPX4^high^ cancers. Thus, the novel findings presented in their study are clinically relevant and have high translational potential.

The mitochondria are major subcellular organelles involved in the production of reactive oxygen species (ROS) and contain a unique system for regulating iron metabolism. Whether mitochondria are involved in ferroptosis has been unclear; however, emerging evidence supports the notion that mitochondrial damage plays an important role in both the initiation and progression of this iron-dependent form of regulated cell death. Previously, we showed that ferroptosis is the major pathogenic mechanism underlying doxorubicin-induced cardiomyopathy in mice by upregulating HO-1 (Heme oxygenase-1), causing the production of excess free iron and oxidized lipids in the mitochondrial membrane. Moreover, we found that mitochondria-targeted therapy significantly reduces ferroptosis-induced cardiac injury.^3^

In addition, the previous study reported that the canonical metabolic activity of mitochondria is required for cysteine deprivation‒induced ferroptosis.^2^ Thus, the findings are consistent with the notion that mitochondria play an essential role in controlling cell fate by regulating ferroptosis.^1^ Importantly, the role of mitochondria in ferroptosis is tightly regulated by the glutathione-dependent enzyme GPX4 and the glutathione-independent enzyme DHODH. Because DHODH couples the pyrimidine biosynthesis pathway to the mitochondrial respiratory chain, inhibiting DHODH should affect respiratory chain function. Several important questions remain, however. First, do similar mitochondrial enzymes regulate ferroptosis by generating CoQH_2_? Second, how is mitochondrial iron homeostasis regulated in order to protect against ferroptosis? Thus, further studies are needed to unravel the full picture by which mitochondria contribute to ferroptosis.

In summary, Mao et al.’s study sheds new light on a novel mechanism by which mitochondria prevent ferroptosis, providing important new preclinical evidence suggesting possible strategies for treating GPX4^low^ cancers by targeting DHODH. Moreover, their study provides a rationale for testing combination therapies in which DHODH inhibitors can be combined with chemotherapy, radiotherapy, and/or immunotherapy, potentially increasing therapeutic efficacy and improving patient outcome. Despite the promising clinical implications with respect to the use of DHODH inhibitors, however, ensuring its safety in children is an important concern, given that DHODH deficiency has been linked to Miller syndrome, a rare genetic condition associated with severe micrognathia, cleft lip, eyelid colobomas, supernumerary nipples, and ulnar ray developmental defects.^4^ Another potential caveat is whether the immune system can affect the efficacy of DHODH-based therapies, as DHODH inhibitors were recently tested in a Phase II trial for autoimmune disease and were shown to have anti‒SARS-CoV-2 and broad-spectrum anti-viral efficacy in vitro.^5^ Nevertheless, future studies are clearly warranted in order to evaluate the feasibility of translating these promising findings into a new strategy for use in the treatment of cancer.
